# Comparative genome analysis of commensal segmented filamentous bacteria (SFB) from turkey and murine hosts reveals distinct metabolic features

**DOI:** 10.1186/s12864-022-08886-x

**Published:** 2022-09-17

**Authors:** Grant A. Hedblom, Kamal Dev, Steven D. Bowden, David J. Baumler

**Affiliations:** 1 Department of Food Science and Nutrition, University of Minnesota-Twin Cities, St. Paul, MN USA; 2 Faculty of Applied Sciences and Biotechnology, Shoolini University, Solan, Himachal Pradesh India; 3 Biotechnology Institute, University of Minnesota-Twin Cities, St. Paul, MN USA; 4 Microbial and Plant Genomics Institute, University of Minnesota-Twin Cities, St. Paul, MN USA

**Keywords:** SFB, Segmented Filamentous Bacteria, Turkey, Comparative genomics, Metabolic capabilities, In Silico

## Abstract

**Background:**

Segmented filamentous bacteria (SFB) are intestinal commensal microorganisms that have been demonstrated to induce the innate and adaptive immune responses in mouse and rat hosts. SFB are Gram-positive, spore-forming bacteria that fail to grow optimally under in vitro conditions due to unique metabolic requirements. Recently, SFB have been implicated in improved health and growth outcomes in commercial turkey flocks. To assess the nature and variations in SFB of turkeys and how they may differ from mammalian-associated SFB, the genome of turkey-associated SFB was compared with six representative genomes from murine hosts using an in silico approach.

**Results:**

The SFB-turkey genome is 1.6 Mb with a G + C content of 26.14% and contains 1,604 coding sequences (CDS). Comparative genome analyses revealed that all the seven SFB strain possesses a common set of metabolic deficiencies and auxotrophies. Specifically, the inability of all the SFB strains to synthesize most of the amino acids, nucleotides and cofactors, emphasizing the importance of metabolite acquisition from the host intestinal environment. Among the seven SFB genomes, the SFB-turkey genome is the largest and contains the highest number of 1,604 predicted CDS. The SFB-turkey genome possesses cellular metabolism genes that are absent in the rodent SFB strains, including catabolic pathways for sucrose, stachyose, raffinose and other complex glycans. Other unique genes associated with SFB-turkey genome is loci for the biosynthesis of biotin, and degradation enzymes to recycle primary bile acids, both of which may play an important role to help turkey associated SFB survive and secure mutualism with its avian host.

**Conclusions:**

Comparative genomic analysis of seven SFB genomes revealed that each strain have a core set of metabolic capabilities and deficiencies that make these bacteria challenging to culture under ex vivo conditions. When compared to the murine-associated strains, turkey-associated SFB serves as a phylogenetic outgroup and a unique member among all the sequenced strains of SFB. This turkey-associated SFB strain is the first reported non-mammalian SFB genome, and highlights the impact of host specificity and the evolution of metabolic capabilities.

**Supplementary Information:**

The online version contains supplementary material available at 10.1186/s12864-022-08886-x.

## Introduction

The gastrointestinal tract of animals harbors a diverse ecosystem of fungi, protozoans, and bacteria, many of which live in mutualistic harmony with their host. The microbial inhabitants of the intestinal mucosa provide numerous benefits to the host organism including synthesis of vitamins and cofactors, metabolizing indigestible polysaccharides and providing protection from colonization by pathogenic bacteria [1, 2]. Additionally, the succession of the gut microbiota is essential in the development and maturation of host innate immunity, impacting the overall composition of cell types present in the host epithelium and thereby alter the intestinal mucosa and the microbiome [3, 4].

Segmented filamentous bacteria (SFB) are host-adapted, intestinal symbionts that influence the adaptive and innate immune responses of their host. SFB are Gram-positive, spore-forming, microaerophilic bacteria characterized by a distinctive filamentous morphology [5–7]. SFB belong to the family *Clostridiaceae* and represent a unique clade of Clostridial cluster 1, also referred to as *Candidatus* Arthromitus, and colonize a broad range of animal hosts, including mice, rats, chickens, turkeys and humans [7–10]. SFB display a unique life cycle involving intestinal binding, filamentation, differentiation, and production of either vegetative or dormant offspring [6]. SFB have been observed to bind to epithelial cells and subsequently induce cytoskeletal rearrangement within the host cell [11]. Once bound to the host epithelium, SFB grow and elongate via the formation of transverse septa to form long filaments of up to 100 µm in length [12]. The ability of SFB to bind to the intestinal mucosa occurs in a host-specific manner, as attempts to inoculate germ-free hosts with SFB homogenates generated from other host species have failed [13].

SFB is one of the first studied examples of a commensal bacterial species that possesses the ability to modulate the adaptive and innate immunity of their host. Intestinal colonization by SFB has been implicated in the production of secretory immunoglobulin A (sIgA), which prevents the colonization of pathogenic microorganisms by blocking epithelial receptors, sterically hindering the ability of pathogens to bind to the mucosa [14, 15]. Additionally, mice mono-colonized with SFB displayed an increased expression of genes commonly associated with inflammation in response to colonization by pathogenic bacteria yet displayed equal fitness to germ-free mice [16].

SFB have been implicated in improved health and growth outcomes in commercial turkey flocks (*Meleagris gallopavo*). In order to investigate the role of microbiome in Light Turkey Syndrome (LTS), Danzeisen et al. performed 16S rRNA analysis microbiome of underweight and normal-weight flocks [10]. Among other symptoms, Turkey poults with LTS weighed 4–5 pounds lighter than the industry average [17]. The comparison of dominant operational taxonomic units (OTUs) between high and under-performing flocks the higher-performing flocks harbored significantly higher proportions of SFBs. This data suggest that SFB may serve as immunostimulatory role in the intestinal mucosa of turkey [10]. The turkey-associated SFB could serve as novel probiotics to ward diseases and lessen the burden of antibiotics usage.

The ex vivo culturing, and optimization of SFB growth outside of a host has not been successful and remained a topic for research to explore its auxotrophic nutritional requirements. During the recent developments, complete genome sequence and metabolomics analysis of turkey-associated SFB have been utilized to predict the metabolic pathways and gain further insights into nutritional requirements [18]. In the current study, we have utilized comparative genomics to compare turkey-associated SFB reported earlier by our research group [18] with mouse- and rat-associated strains of SFB previously sequenced and analyzed [19–22]. The focus of earlier studies has been to analyze the genomes of SFB strains for their metabolic capabilities, host-adaptive factors, and flagellar genes. There has been little research on comparative genomics to compare and assess the diverse metabolic potential to address the issues of adaptation and divergence.

In the current study, we have identified a set of core metabolic determinants shared among seven SFB strains and genetic traits essential for microbe-host interactions. It is interesting that turkey-associated SFB are very distinct from the murine-associated SFB strains. Turkey-associated SFB possess several unique metabolic characteristics including an increased capacity to degrade amino acids (serine, threonine, glutamine), metabolize a broader array of mono/disaccharide substrates, break down complex glycans, hydrolyze and recycle primary bile acids, and synthesize, rather than degrade biotin. Differences in these strains may provide further insights into better understand the growth requirements under in vitro conditions, develop probiotic formulations for turkey poults, and molecular basis of coevolution in a given host organism.

## Results

### General features of the turkey-associated SFB genome

**Fig. 1:**
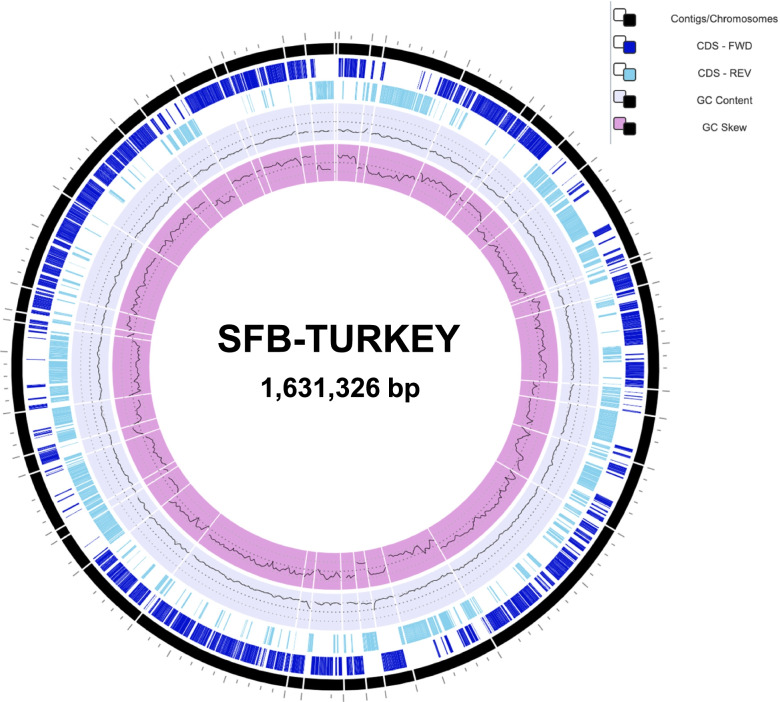
Circular map of the SFB-turkey genome. The 41 contigs were arranged into a circular pseudochromosome (see Materials and methods). Circles from the outside in are as follows: (1) Contig mapping including assembly gaps. (2) Coding Sequences (CDS) on the forward strand of the chromosome. (3) Coding Sequences (CDS) on the reverse strand of the chromosome. (4) G + C content of each loci on the bacterial chromosome. (5) G + C skew at each loci on the bacterial chromosome

The turkey SFB draft genome “*Candidatus* Arthromitus UMNCA01” has been published and can be accessed via the GenBank accession number GCA_001655775.1 [18], henceforth referred to as SFB-turkey, and consists of a single circular chromosome with 1,631,326 base pairs and assembled into 41 contigs (Fig. 1). The single chromosome possesses an average G + C content of 26.14%, which is slightly lower than other members of Clostridial Cluster I [20]. A total of 1,604 coding sequences (CDS) were predicted from the SFB-turkey genome sequence, with an average length of 931 base pairs (Table 1). The genome sequence was annotated using the National Center for Biological Information (NCBI) Prokaryotic Genome Annotation Pipeline and subsequently analyzed using the Pathosystems Resource Integration Center (PATRIC) [23]. Of the predicted 1,604 CDS, 1,035 CDS were assigned functional roles using PATRIC, representing a 64.5% coverage of the predicted CDS, comparable with the genomes of SFB strains (Table 1). The remaining 569 CDS were not assigned any functional roles and were instead designated as hypothetical proteins.

**Table 1:** Genome features of seven strains of segmented filamentous bacteria and *Clostridium beijerinckii*

Genomic features	SFB-Turkey	SFB-Mouse-Japan	SFB-Mouse-YIT	SFB-Mouse-NYU	SFB-Mouse-NL	SFB-Mouse-SU	SFB-Rat-YIT	*Clostridium beijerinckii* NRRL B-593
Genome Size (bp)	1,631,326	1,620,005	1,586,397	1,569,870	1,654,902	1,566,160	1,515,556	6,156,662
G + C Content (%)	26.14	28.26	28.11	27.90	28.10	27.80	27.98	29.57
CDS	1,604	1,540	1,499	1,503	1,594	1,511	1,412	5,808
tRNAs	33	38	37	36	37	37	37	52
rRNAs	1	18	15	6	15	17	16	3
Hypothetical Proteins	569	442	421	426	519	422	379	1,854
Proteins with Functional Assignments	1,035	1,098	1,078	1,077	1,075	1,089	1,033	3,954
Proteins with Functional Assignments (%)	64.53%	71.29%	71.91%	71.66%	67.44%	72.07%	73.16%	68.08%

The SFB-turkey genome contains a single rRNA operon and 33 tRNA genes, a relatively low number of rRNA operons as compared to other members of Clostridial cluster I (3) and among SFB representatives (6–18) (Table 1) [21]. We believe this low number of rRNA operons identified in the SFB-turkey genome is likely a result of the draft status of the SFB-turkey genome, due to the repetitive sequence region of rRNA. A single CRISPR sequence with 11 identical repeats and 10 unique spacer sequences was identified, along with 7 CRISPR-associated (Cas) proteins (including Cas1-Cas5), which are localized on contig 38. CRISPR sequences and associated proteins confer resistance to exogenous genetic elements [24]. The presence of these CRISPR loci suggests that SFB are frequently exposed to foreign invading mobile genetic elements such as bacteriophages.

### Comparative functional genomic characteristics of seven SFB strains

Six representative SFB genomes from murine hosts (5 from mouse hosts and 1 from rat) were selected (GCA_000284435.1, GCA_000283555.1, GCA_000270205.1, GCA_000709435.1, GCA_000225365.2, GCA_000252785.2) to compare and assess the functional characteristics of turkey-derived SFB. Although 10 additional genomes of SFB are available in the PATRIC database, the host the strains were isolated from was not provided, therefore we focused on comparative analysis of six other SFB genomes isolated from specific hosts for the comparative analysis to identify genome differences that may be related to SFB strains host-specificity. The annotated genome sequences of SFB-mouse-Yit, SFB-mouse-Japan, SFB-mouse-NYU, SFB-mouse-NL, SFB-mouse-SU, SFB-rat-Yit, and SFB-turkey were selected and analyzed using PATRIC analytical tools (Methods). The SFB-turkey genome was analyzed using the PATRIC similar genome finder (Methods) to find the most closely related non-SFB, free-living bacteria for phylogenetic comparisons.

*Clostridium beijerinckii* strain NRRL B-593 was identified as the most closely related, non-SFB, bacterial strain to SFB-turkey, therefore have included *C. beijerinckii* as a comparative outgroup for the comparison of SFB gene number and subsystems content, since *C. beijerinckii* is the closet free living bacteria available in the PATRIC database for usage in this analysis. Evolutionarily it is known that host-dependent bacteria lose regions of the genome due to the unnecessary need for production of all metabolic enzymes, metabolites, and biological building blocks due to availability from the host environment, therefore this information aids to understand the limitations and scant metabolic capabilities of the host-dependent SFB strains genomes in comparison to a free-living bacteria capable of living and replicating outside of a host. we used *C. beijerinckii* as a Clostridial reference genome to study the general features of murine and turkey SFB strains. Though not closely related to the murine and turkey SFB genomes, *C. beijerinckii* remained the closest relative based on the similar genome finder predictions and was therefore used as a phylogenetically related outgroup.

When comparing the genome sequence of all the selected murine and turkey SFB strains, the SFB-turkey genome was predicted to possess the highest number of CDS (1604) and second lowest number of proteins with assigned functions (1035) (Table 1). The SFB-turkey genome is the second largest SFB genome and is bigger than the average genome size of mouse associated SFB. SFB-turkey possesses a G + C content of 26.14%, which is roughly 2% less than the murine genomes, and around 3.5% shorter than that of *C. beijerinckii* (Table 1). The divergence in G + C content indicates that SFB-turkey is the most evolutionarily divergent and dissimilar strain among all of the sequenced genomes of murine and the turkey-SFB strain (Fig. 1). The overall genome size of *C. beijerinckii* is approximately 3.8 times larger than the average SFB genome size and is similarly predicted to possess 3.8 times more CDS (5,808) than SFB (Table 1).

**Fig. 2:**
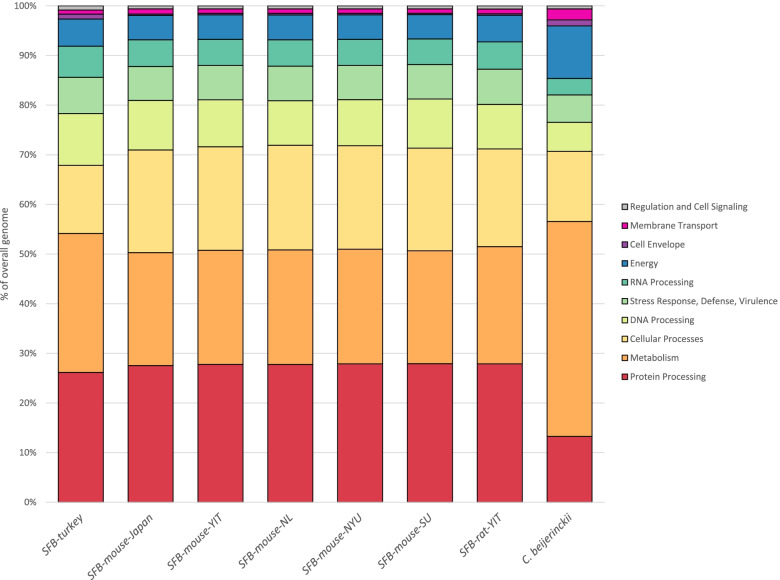
Subsystems assignments of seven strains of segmented filamentous bacteria and *Clostridium beijerinckii*. Overview of the PATRIC Subsystem assignments of the seven analyzed genomes of SFB strains and *Clostridium beijerinckii* as represented by the percent of genes in each Subsystem category

To assess the overall functional distribution of the analyzed SFB genomes, we compared PATRIC Subsystems assignments for each of the murine and turkey SFB strains and *C. beijerinckii* NRRL B-593. On an average, around 25% of all the genes (158) with subsystems assignments in SFB are involved in protein processing, while only 13% genes (224 genes) of *C. beijerinckii* were assigned functions that are involved in protein processing (Fig. 2, Figure S1). The genomes of analyzed SFB strains are deficient in metabolism subsystems, which comprised an average of 24% (155 genes) of the assigned SFB genes, and 43% (731 genes) of the assigned genes in *C. beijerinckii*. The genomes of murine-associated strains of SFB appear fairly consistent in the distribution of subsystems, while SFB-turkey possesses a greater proportion of genes associated with metabolism and a smaller proportion of genes associated with cellular processes, such as cell division, sporulation and chemotaxis, than the murine-associated strains. In the “metabolism” category, SFB-turkey genome encodes more enzymes (169 genes) than the murine SFB strains(155 genes) (Figure S1). Conversely, within the “cellular processes” category, SFB-turkey is predicted to encode fewer proteins (83) than the average of the murine SFB strains (155). The CDSs for other subsystems categories, such as DNA processing, RNA processing, energy production and associated pathways are very conserved across all the murine and turkey-SFB genomes. Comparison of the subsystems assignments of SFB turkey with *C. beijerinckii* showed that SFB have encodes for fewer genes in every subsystems category (Table S1, Figure S1), but the magnitude of these differences is not consistent across all the subsystems. For example, *C. beijerinckii* is predicted to possess approximately 1.25 times more genes involved in protein processing than the turkey SFB, but are predicted to possess five times more genes involved in metabolic functions (Table S1). These results highlight that SFB strains are highly dependent on the host system and lacks very crucial pathways such as de novo synthesis of amino acids, nucleotides and cofactors. These findings serve to highlight the overall auxotrophic nature of SFB and defines their phylogenetic position between free-living bacterial and obligate intracellular symbionts [19].

**Fig. 3:**
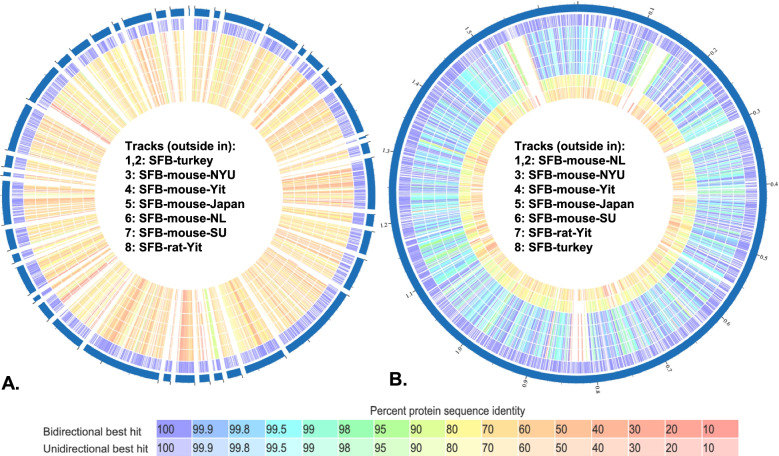
Proteome comparison of seven strains of segmented filamentous bacteria. Protein sequence-based genome comparison of genomes of seven SFB genomes using bidirectional BLASTP using SFB-turkey as a reference genome (**A**), or SFB-mouse-NL as a reference genome (**B**)

While examining the phylogenetic position of SFB-turkey in relation to the murine-associated strains of SFB, it was observed that SFB-turkey is dissimilar to the murine-associated SFB strains and serves as an outgroup in a phylogenetic tree (Figure S2). All the mouse-associated SFB strains are phylogenetically closely related, with SFB-rat-Yit serving as the most dissimilar among rodent-associated strains of SFB. The overall protein sequence similarity analysis showed that SFB-turkey has around 70% similarity with murine-associated SBF strains (Fig. 3). The mouse-associated SFB strains display far greater protein sequence homology to each other (avg around 99.5%), and are much more dissimilar to the sequences of SFB-rat-Yit and SFB-turkey (Fig. 3). These findings further underscore the role of host-association on the evolutionary divergence of SFB.

### Core Metabolic Characteristics of SFB genomes

**Fig. 4:**
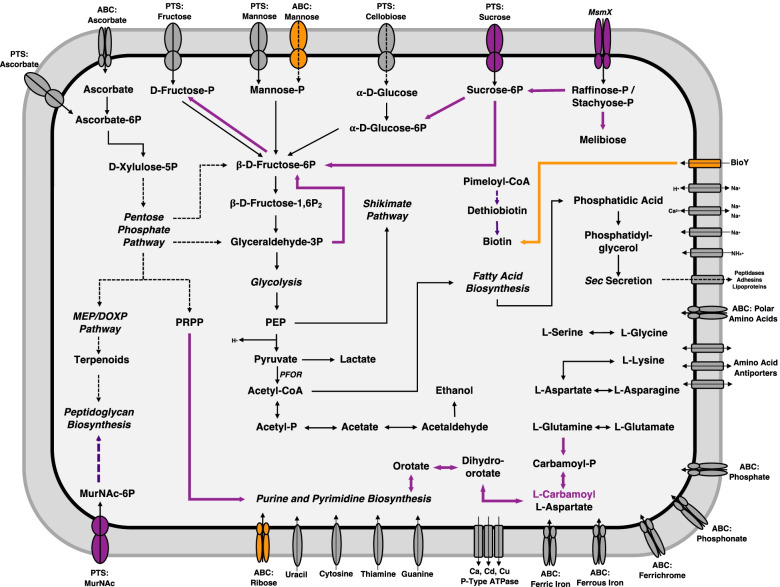
Comparative metabolic pathways between SFB-turkey and murine SFB strains. A schematic comparison of the metabolic capabilities of SFB strains isolated from turkey and murine hosts. Color coding for divergent metabolism is as follows: enzymatic pathways and transporters that are shared by all SFB strains appear in black, complete metabolic pathways conserved in each SFB strain appear in bold and italics, enzymatic pathways and transporters that are unique to SFB-turkey appear in purple, enzymatic pathways and transporters that are unique to the murine-associated strains appear in orange. Oval shapes are used to indicate permeases, rectangles represent import/export-type transporters. Contiguous lines represent single enzymatic reactions, while dashed lined represent a series of conserved enzymatic pathways

The genome of the seven analyzed SFB strains possesses a core set of metabolic capabilities and deficiencies. The genome of each SFB strain exhibits several common features of some endosymbiont anaerobic bacteria, lacking any CDS for the electron transport chain and tricarboxylic acid cycle. The genomes of SFB strains are predicted to possess all the enzymes of the glycolytic pathway for the conversion of glucose to pyruvate and the non-oxidative phase of the pentose phosphate pathway. In contrast, glucose-6-phosphate dehydrogenase, gluconolactonase, and 6-phosphogluconate dehydrogenase enzymes of the oxidative phase of pentose phosphate pathway were absent. As expected, the genome of each SFB strain possesses pathways for the production of lactate, alcohol and acetate from pyruvate and hence, likely to generate energy through anaerobic fermentation (Fig. 4). In addition, the genomes of all the SFB-strains encode genes for alcohol, aldehyde, and lactate dehydrogenases, which facilitate the oxidation of substrates in the fermentation process. The action of pyruvate ferredoxin oxidoreductase generates acetyl-CoA, carbon dioxide and hydrogen ions; the latter is likely to be used by SFB to create a proton gradient for ATP synthesis [20]. SFB likely to utilize fermentation byproducts for substrate-level phosphorylation via the action of phosphoglycerate kinase, acetate kinase, and pyruvate kinase in a method similar to that of other *Clostridia* [25]. Each SFB strain encodes genes for catalases and one peroxidase, indicating that SFB are likely to exhibit some degree of oxygen tolerance in the microaerobic environment of the host intestine.

SFB rely on their host and the surrounding intestinal environment for the uptake of numerous carbohydrates, which is supported by the presence of several carbohydrate transporters. Further, genes encoding several phosphotransferase systems (PTSs) and ATP-binding cassette (ABC) transporters for the uptake of various carbohydrates, including ascorbate, fructose, glucose, mannose, mannitol, and cellobiose were predicted in the genomes of all the selected SFB strains (Fig. 4). Additionally, the complete enzymatic pathways to convert these substrates to glyceraldehyde-3-phosphate were also detected. The complete enzymatic suite of the *ula* operon containing the genes for the production of enzymes for the transport and conversion of L-ascorbate to D-xylulose-5-phosphate, which is later used in the non-oxidative phase of the pentose phosphate pathway was predicted to be encoded by the genomes of all the representative SFB strains. The genomes of all the SFB strains are predicted to participate in the foraging of glycans from the intestinal epithelium, encoding enzymes involved in the import and utilization of mannose, N-acetylglucosamine, and sialic acid. To degrade these complex host glycans, SFB secrete alpha-N-acetylglucosaminidase and endo-β-N-acetylglucosaminidase. Each of the SFB strains have Sec-dependent secretory machinery; encoding for SecA, SecD, SecE, SecF, SecG, and SecY. Similar to previous analyses, the translocation-specific chaperone SecB appears to be absent in the genomes of SFB strains, suggesting that SFB utilize a modified Sec system [20].

Each of the analyzed SFB genomes is predicted to lack many critical enzymes for the biosynthesis and catabolism of amino acids, and instead likely rely on the digestion and uptake of exogenous peptides for amino acid acquisition. The SFB genomes only encode enzymes for the synthesis of lysine and for the interconversion of aspartate, glutamate, asparagine, and glutamine. Additionally, glycine hydroxymethyltransferase catalyzed interconversion reactions of glycine and serine were predicted in the genomes of all the SFB strains. Despite the lack of encoded enzymes for the production of amino acids, several permeases and transporters for the uptake of amino acids and oligopeptides were detected in the genomes of SFB strains, indicating an efficient system for the uptake of amino acids from the host. To breakdown the exogenously acquired oligopeptides to utilizable amino acids, the genomes of all the SFB strain encodes numerous peptidases and proteases. The genomes of all the SFB strains either showed incomplete or completely absent biosynthetic pathways of most essential vitamins and cofactors (such as B1, B2, and B12, pyridoxine, nicotinamide, pantothenate, and biotin). Also, the genomes of SFB strains are unable to synthesize nucleotides independently; instead utilizing alternative pathways that rely largely on the exogenous uptake of nucleotide bases via the use of several PTSs and other permeases (Fig. 4).

The genomes of all the SFB strains possess complete enzymatic pathways for the shikimate shunt, the synthesis but not degradation of fatty acids, and the non-mevalonate pathway (MEP pathway) for the synthesis of terpenoids that are involved in the synthesis of peptidoglycan (Fig. 4). In order to compete with the host and members of the intestinal microbiota for iron acquisition, the genomes of SFB strains encode a number of iron transporters and uptake systems. Each strain encodes ATP-binding cassette proteins for the uptake of ferric iron, ferrous iron, and ferrichrome (Fig. 4). The uptake of these siderophores is essential, since all the SFB strains lack the ability to synthesize siderophores of their own.

### Unique host adaptation/specificity characteristics of turkey-associated SFB

Despite a broad array of common characteristic features among the genome sequence of six murine and one turkey-associated SFB, there are several unique characteristics of SFB-turkey that make this strain distinct from the murine-associated SFB strains. In considering the ability of each strain to metabolize carbohydrates, the genome of SFB-turkey is predicted to encode more enzymes for the uptake and utilization of a broader array of substrates. Unlike the six murine genomes, we observed that the genome of SFB-turkey is predicted to encode all the enzymes of the pentose phosphate pathway. Specifically, transaldolase is the enzyme of this pathway that is only present in the turkey-SFB genome and catalyzes the reaction of sedoheptulose 7-phosphate and glyceraldehyde 3-phosphate to produce erythrose 4-phosphate and fructose 6-phosphate (Fig. 4). The genome of SFB-turkey is also predicted to encode the fructokinase enzyme, that catalyzes the transfer of a phosphate group from ATP to fructose as a first step in its utilization in glycolysis.

The host intestinal environment contains a variety of bioavailable glycans, either from the host itself or from the intestinal microbiota. Like many other mucosa-associated bacteria, SFB must first cross the mucus layer that serves as a protective barrier to prevent other bacteria from reaching the epithelium. It has been previously reported that SFB’s have the ability to utilize certain monosaccharides, such as mannose and N-acetylglucosamine, that provide a competitive advantage in intestinal colonization [21]. Unlike the murine-associated strains of SFB, SFB-turkey is predicted to utilize sucrose, raffinose, stachyose, and sialic acid as potential carbohydrate sources. SFB-turkey is also predicted to encode the enzyme beta-fructosidase (invertase), which catalyzes the hydrolysis of terminal non-reducing beta-D-fructofuranoside residues leading to the conversion of sucrose to D-fructose and D-glucose, raffinose to melibiose and D-fructose, and stachyose to manninotriose and D-fructose (Fig. 4). To import these carbohydrates, only the SFB-turkey genome encodes a putative phosphotransferase system for the uptake of sucrose and the maltodextrin ABC transporter (*MsmX*). The presence of these enzymes specifically in the SFB-turkey genome suggests that it has developed a competitive advantage in comparison to murine SFB to adapt to a highly complex host intestinal environment in order to utilize intermediate glycans as primary carbohydrate sources.

In considering the ability of each strain to synthesize and utilize purines and pyrimidines, SFB-turkey is distinct from the murine-associated SFB strains. The genome of SFB-turkey encodes for the metabolic pathway for the de novo synthesis of purine nucleotides from phosphoribosyl pyrophosphate (PRPP) through the action of uracil phosphoribosyltransferase, suggesting that turkey-associated SFB alone utilize pentose phosphate pathway products in the biosynthesis of nucleotides (Fig. 4, Figure S3) [26]. The genome of SFB-turkey also possesses a pathway for the conversion of L-glutamine to orotidine-5-phosphate, which is involved in the aforementioned pathway for the synthesis of nucleotides from PRPP. This pathway represents the only example in the examined SFB genomes that utilizes amino acids as an intermediate for the synthesis of nucleotides and may underscore an evolutionary divergence node between SFB-turkey and the murine SFB strains.

In addition to the aforementioned differences in carbohydrate and nucleotide metabolism, SFB-turkey is the only SFB strain predicted to be able to synthesize biotin. SFB-turkey has been predicted to encode 8-amino-7-oxononanoate, adenosylmethionine-8-amino-7-oxononanoate transaminase, dethiobiotin synthase and biotin synthase, the key enzymes for the biosynthesis of biotin. In contrast, murine-associated SFB strains are predicted to lack all the enzymes involved in synthesis of biotin. To compensate for the lack of biosynthetic pathways for the production of biotin, murine-associated strains are predicted to encode the BioY biotin ECF transporter, which is likely to be involved in the import of biotin into the cell and its subsequent metabolism. Interestingly, SFB-turkey genome is also the only analyzed strain not predicted to encode the BioY biotin ECF transporter. Each SFB strain is predicted to encode all the subunits of the biotin-ligase holoenzyme for the conversion of biotin into biotinyl-CoA and holocarboxylase, which are involved in the production of urea and fatty acid biosynthesis respectively.

Another major difference between the genomes is that SFB-turkey possesses genes that encode enzymes for the hydrolysis of primary bile acids, that are absent from the murine SFB genomes. SFB-turkey is predicted to encode choloylglycine hydrolase (bile salt hydrolase), which allows for the production of taurine, cholate, glycine and chenodeoxycholate from the conjugated bile salts. With the exception of glycine, SFB-turkey has no apparent enzymatic steps or pathways to utilize these secondary bile acids and their precursors. Though this enzyme was previously predicted to be encoded by SFB-mouse-SU [22], but our analysis predicted it to be only encoded by SFB-turkey. Bile acids have been reported as immunomodulatory and possess antimicrobial activities [27], and this may require SFB-turkey to synthesize bile acid hydrolase to overcome the antimicrobial effects of these host factors in the avian ileum. This mechanism has been reported in other gut commensals [28]. Deconjugated bile salts are less efficiently absorbed and more likely to be excreted by the host, increasing the demand for cholesterol for de novo synthesis of bile acids to replenish the loss of bile salts [29]. In mouse studies, comparison of mice inoculated with strains of *Bacteroides* with and without bile salt hydrolases, it was found that the mice colonized with bile salt hydrolase-deficient bacteria gained less weight and had lower levels of fats and cholesterol in their blood and liver and tend to metabolize fats rather than carbohydrates to generate energy [30].

Finally, SFB-turkey was the only SFB strain predicted to encode the subunits of a phosphotransferase system for the import and phosphorylation of *N*-acetylmuramic acid and *N*-acetylmuramic acid-6 phosphate etherase for the conversion of *N*-acetylmuramic acid-6 phosphate to N-acetylglucosamine-6-phosphate (Fig. 4). N-acetylglucosamine-6-phosphate is used as an intermediate in the production of the host peptidoglycan layer and allows SFB-turkey to utilize alternate pathways to synthesize the cell wall of these bacteria.

### Genes involved in sporulation and motility

According to the PATRIC subsystem assignments of each of the SFB genomes, each of the examined SFB strains encodes several enzymes involved in sporulation and germination. Key genes encoding sporulation-related sigma factors were identified in each of the SFB strains. Like many other closely related *Clostridia*, each SFB strain lacks the essential enzymes involved in the phosphorelay system for sporulation initiation, such as Kin, Spo0B, and Spo0F [20]. The genomes of all the SFB strains are predicted to encode the same set of sporulation genes, with the exception of *SpoIIR*, a gene required for processing and compartmentalization of pro-SigE, which is not encoded by SFB-turkey. The genomes of all the murine and turkey-SFB encodes a single *ger* operon, which contains germinant receptors belonging to the GerA family. In the genome of each SFB-strain, the *ger* operon is directly preceded by *cphAB* and *ispE* genes upstream of the operon. The *cphAB* genes encode for cyanophycin synthase and cyanophycinase, which are necessary for the biosynthesis of cyanophycin, an amino acid polymer comprised of Aspartic acid and Arginine [20]. Cyanophycin may be used by germinating SFB spores as an amino acid source during the process of spore germination. The *ispE* gene encodes 4-diphosphocytidyl-2-C-methyl-D-erythritol kinase, which is a necessary enzyme in the methyl-erythritol phosphate (MEP) pathway for the synthesis of terpenoids [20]. Terpenoids are essential components in peptidoglycan and cell membrane synthesis. The proximity of these gene loci to the *ger* operon suggests that the synthesis of these enzymes may be coupled with spore germination and transcribed in tandem with the operon.

The genomes of the murine-associated SFB strains encode 32 genes for the synthesis and assembly of flagella, whilst the turkey-associated SFB is predicted to encode only 29 genes for the synthesis and assembly of flagella. This include genes encoding for the flagellin subunit FliC, as previously predicted in the genome sequences of SFB strains [19, 20]. Each SFB strain encodes at least two copies of the *fliC* gene, with SFB-turkey encoding 4 copies. Flagellins are key filament proteins that have been implicated in the innate production of Th17 cells, serving as an agonist of Toll-like receptor 5 (TLR5) in intestinal dendritic cells and triggering the NF-κB signaling pathway that leads to cytokine production [31, 32]. The presence of a complete flagellar apparatus in most SFB strains suggests that these organisms may be motile at some point in the during the life cycle of SFB. SFB may also use flagella for penetrating the intestinal mucosa in a method similar to that of other intestinal bacteria [33]. Despite the proposed function of flagella in cellular motility, flagellated SFB cells have yet to be observed microscopically. Unlike the murine-associated strains of SFB, the SFB-turkey genome lacks the genes encoding FliL, FlgN, and FlbD, which are responsible for flagellar rotation, flagellar assembly initiation, and transcriptional regulation, respectively [34]. The absence of these aforementioned genes may indicate that SFB-turkey is the only strain with an incomplete flagellar apparatus and perhaps lost the ability to utilize this structure for motility and chemotaxis related functions. The flagella in SFB-turkey may only serve as a surface antigen to present to TLR5 in the intestinal epithelia, losing the functionality of the complete flagellar apparatus during evolution. However, the perceived absence of these genes in the SFB-turkey genome may be due to low sequence homology of predicted CDS to known proteins. Finally, each SFB strain is predicted to encode 8 chemotaxis related proteins, including CheY, which is involved in transmitting chemoreceptor signals directly to the flagellar motor complex [34].

## Discussion

The whole-genome sequence of SFB-turkey and its comparison with several reported murine SFB strains, revealed a number of conserved characteristics of all segmented filamentous bacteria, and several metabolic capabilities that are unique to SFB-turkey. All SFB have a highly reduced genome size in comparison to other free-living bacterial genomes and are deficient in pathways for the de novo biosynthesis of amino acids, cofactors and nucleotides, thus underscoring the auxotrophic nature and non-culturability of this unique group of *Clostridia*. To counteract these metabolic deficiencies, each SFB strain encodes numerous proteases, peptidases, permeases, and transporters to aid in the digestion and uptake of exogenous amino acids and nucleotides that are present in the host intestinal epithelia, capitalizing on the bioavailability of host or microbiome factors for survival and functionality. Due to the absence of core metabolic pathways, all the SFB strains are not tractable to culture under laboratory conditions. Such genome analysis and comparison could help in providing insights in the development of growth media suitable for optimal growth under in vitro conditions and their evolutionary strategies.

The analyses performed in the current study have a few limitations. Not every genome analyzed in this study is a complete genome, with the genomes of SFB-turkey, SFB-mouse-NYU, and SFB-mouse-SU serving as draft genomes. These draft genome sequences are incredibly valuable, as most genes, their classifications, and their relatedness to the genes of other organisms are represented in these sequences. However, these draft assemblies have the potential for errors caused by collapsed repeats, rearrangements, and inversions; as well as having an unknown portion of the genome unaccounted for in assembly gaps. These unaccounted-for gaps may contain repeated elements such as rRNA operons, and regions which function in controlling DNA rearrangements via chromosomal deletions, duplications, and inversions which may be missed unless contig gaps are closed and the draft genome is completed. Completion of the SFB-Turkey genome would likely identify additional rRNA operons and IS regions not present in the draft version, but would likely not change the analysis of the genes involved in the metabolic and bacterial host interaction content.

SFB thrive in the host intestinal epithelium, an ecological niche rich in simple carbohydrates produced by the degradation of complex carbohydrates by brush-border digestive enzymes. SFB likely require these monosaccharides and disaccharides to survive, as they lack the enzymes for the digestion of more complex glycans and polysaccharides. However, unlike the murine-associated strains of SFB, turkey-associated SFB is predicted to encode a much more diverse set of enzymes for the degradation of carbohydrates, including invertase, which allows this strain to utilize sucrose, raffinose, and stachyose as potential carbohydrate sources. The ability of SFB-turkey to utilize a broader array of carbohydrates and glycans than their murine-associated counterparts may indicate an evolutionary divergence due to the dietary difference and intestinal microbiome compositions of commercial turkey in comparison to SFB of mice or rats. Additionally, turkey-associated SFB utilize amino acids and nucleotides in a manner distinct to that of murine-associated SFB strains. Unlike the murine-associated strains of SFB, turkey-associated SFB are predicted to synthesize biotin, circumventing a key metabolic deficiency of its murine-associated SFB counterparts. SFB-turkey was the only strain predicted to encode choloylglycine hydrolase to deconjugate primary bile acids, allowing the SFB-turkey strain to avoid the antimicrobial action of these compounds and may potentially serve to benefit the avian host through lowering host cholesterol, warranting further study and examination. These observed divergences in metabolic capabilities suggest that each member of this group of bacteria has developed host-adapted capabilities to best associate with their host and the gut microbiome therein, securing mutualism and survival. In addition to the immunostimulatory effects of SFB on the stimulation of Th17 cell development and immunoglobulin production, the presence of choloylglycine hydrolase may also serve to improve performance outcomes of commercial flocks in the prevention of Light Turkey Syndrome.

In conclusion, our work provides important evidence suggesting that segmented filamentous bacteria isolated from evolutionarily divergent host organisms possess distinct enzymatic pathways for carbohydrate, nucleotide, amino acid, and vitamin and cofactor metabolism, indicating the role of host-adaptation in securing a mutualistic relationship with their host. Although, this analysis is highly dependent on gene annotation comparison to distantly related species, as more genomes of closely related species and strains are sequenced and gene function determined, future analysis may reveal additional differences of gene content related to metabolic capabilities and host-interaction. In addition, this analysis relies on gene annotation and pathway completeness or incompleteness to describe whether a strain is expected to catabolize a given compound or synthesize others, and future studies such as the use of genome-scale metabolic models are an approach to elucidate more detail of the metabolic capabilities of catabolism of SFB strains. The core metabolic deficiencies of each SFB strain highlighted in our study will aid in future attempts to cultivate these bacteria from other host organisms and help in developing probiotics for dairy and poultry industries.

## Materials and methods

### Genome sequencing and assembly of SFB-turkey

*Candidatus* Arthromitus UMNCA01 strain of SFB was isolated from the gut microbiome of commercial turkey raised in a research flock at the University of Minnesota. The UMNCA01 draft genome has been published and can be accessed via the GenBank accession number GCA_001655775.1 [18]. Sequence data were assembled using CLC Genomics Workbench v. 9.0/APRIL-2016, with default parameters, and then contigs were mapped to an existing mouse “Candidatus Arthromitus” genome using Mauve [35] to retrieve and arrange Turkey-SFB sequences that mapped to those genomes. Following manual curation, unmapped contigs were then filtered from the metagenomic assembly. The genome sequence of “Candidatus Arthromitus” UMNCA01 was annotated using the National Center for Biological Information (NCBI) Prokaryotic Genome Annotation Pipeline and best-placed reference protein set of GeneMarkS+ (Annotation Software version 4.6) as described [18]. The genome sequence and global statistics of UMNCA01 genome have been recently published by Hedblom et al. [18].

### Genomes of SFB bacteria used in current study

The strains used in this study represent assembled SFB genomes of mouse, rat and turkey hosts are as follows: SFB-mouse-YIT (GCA_000284435.1), SFB-rat-YIT (GCA_000283555.1), SFB-mouse-Japan (GCA_000270205.1), SFB-mouse-NL (GCA_000709435.1), SFB-mouse-NYU (GCA_000225365.2), SFB-mouse-SU (GCA_000252785.2), and SFB-turkey (GCA_001655775.1).

### Assessing functional similarity of SFB genomes

The genomes of seven murine and turkey SFB were compared and analyzed at the level of genes and protein sequences using the Pathosystems Resource Integration Center (PATRIC) Bioinformatics Resource Center [23]. To compare the functional distribution of the contents of the SFB genomes, we used the PATRIC Subsystems analysis tool. Subsystems, like clusters of orthologous genes are collections of functionally related proteins that are enumerated and divided into superclass (example: metabolism), class (example: biotin metabolism), subclass, and subsystem [36, 37]. In order to enable comparative analyses of eight genomes and original annotations using consistent vocabulary, all the genomes were annotated using a customized version of the RAST tool kit (RASTtk) generated by the PATRIC Genome Annotation service [37]. Each genome was annotated concurrently using the same version of the RASTtk.

PATRIC assigns protein encoding genes protein family membership in order to drive comparative analysis tools and KEGG metabolic pathway information [38].

### Detection of differential metabolic pathways among genomes of SFB

In order to compare the presence and absence of proteins and specific metabolic pathways, the PATRIC Proteome Comparison tool, which uses a bidirectional best BLASTP analysis, was implemented [39]. In order to sort and compare these protein families, PATRIC Cross-Genus Protein Families (PGfams) were used [40]. PGfams are comparable clusters of proteins that likely have been assigned similar functional assignments and annotations. For proteins with no annotation, BLAST similarity search was used to cluster these sequences [39]. The PGfams clusters were used for cross-genus comparison due to their slightly relaxed clustering criteria, which was selected for the relative novelty and scarcity of SFB genomes. The Proteome Comparison tool allowed for the filtering of functionally assigned gene families based on the presence or absence of these genes among all the selected genomes. This tool was used to examine functional families that were specifically present or absent only in SFB-turkey.

### Sequence similarity and phylogenetic tree construction

To determine the sequence similarity and phylogenetic relationship between the genomes of SFB, the PATRIC Similar Genome Finder was implemented [23]. The Similar Genome Finder utilizes Mash, which functions by reducing whole genome sequences into representative sketches that are used for estimating mutation rates of analyzed k-mer sequences [41]. This tool compares against all public genome sequences and yields the distance between these sequences and a corresponding *P*-value [42]. For phylogenetic tree construction, the genomes of SFB strains were analyzed through the PATRIC Codon Trees pipeline, which utilizes PATRIC Cross-Genus Protein Families (PGfams) to align 1000 single copy protein and nucleotide sequences using MUSCLE and Biopython respectively [43, 44]. Support values for the phylogenetic tree were generated from 100 rounds of rapid bootstrapping in RAxML [45]. The *P*-value threshold used was 0.001, and the maximum distance between sequences was set at 0.5 to allow for SFB-rat and SFB-turkey to be included.

## Supplementary Information

**Additional file 1: Table S1.** Numerical Subsystems Assignments of seven strains of segmented filamentous bacteria and Clostridium beijerinckii. Number of genes in each PATRIC Subsystem category of the seven analyzed SFB strains and Clostridium beijerinckii.

**Additional file 2: Figure S1.** Averaged Subsystems Assignments of seven strains of segmented filamentous bacteria and Clostridium beijerinckii. PATRIC Subsystem assignments of the seven examined SFB strains and Clostridium beijerinckii as represented by the number of genes in each Subsystem category. Each of the 5 mouse-associated strains have been averaged and are represented as SFB-mouse (avg).

**Additional file 3: Figure S2.** Phylogenetic Tree of seven SFB strains. Phylogenetic tree representing all the seven genomes of SFB strains. Tree was assembled using the PATRIC Codon Trees pipeline.

**Additional file 4: Figure S3.** Comparative Nucleotide Metabolism Pathways Between SFB-turkey and Murine SFB Strains. A schematic comparison of metabolic capabilities of SFB strains isolated from turkey and murine hosts to synthesize and degrade purines and pyrimidines. Color coding for divergent metabolism is as follows: enzymatic pathways shared by all the SFB strains appear in black, enzymatic pathways and transporters that are unique to SFB-turkey appear in purple, and complete metabolic pathways conserved in each strain appear in bold and italics.

## Data Availability

The datasets utilized in the present study are available in the National Center for Biology Information (NCBI) [https://www.ncbi.nlm.nih.gov/], GenBank repository, under accession numbers as GCA_000284435.1, GCA_000283555.1, GCA_000270205.1, GCA_000709435.1, GCA_000225365.2, GCA_000252785.2, and GCA_001655775.1. Additional results supporting findings presented in this study are provided in the supplementary information section.

## Abbreviations

SFB: Segmented filamentous bacteria
CDS: Coding sequences
PATRIC: Pathosystems Resource Integration Center
CRISPR: Clustered regularly interspaced palindromic repeats
PTS: Phosphotransferase system
ABC: ATP-binding cassette
TLR5: Toll-like receptor 5
BLAST: Basic Local Alignment Search Tool
LTS: Light Turkey Syndrome
