# Supplementary material for: Comparative genome analysis of commensal segmented filamentous bacteria (SFB) from turkey and murine hosts reveals distinct metabolic features
Source: BMC Genomics. 2022 Sep 17;23:659. doi: 10.1186/s12864-022-08886-x (PMC9482736; doi:10.1186/s12864-022-08886-x)
Supplement: Supplementary file 1 — Additional file 1: Table S1. Numerical Subsystems Assignments of seven strains of segmented filamentous bacteria and Clostridium beijerinckii. Number of genes in each PATRIC Subsystem category of the seven analyzed SFB strains and Clostridium beijerinckii. [file 12864_2022_8886_MOESM1_ESM.pptx]

## Slide 1
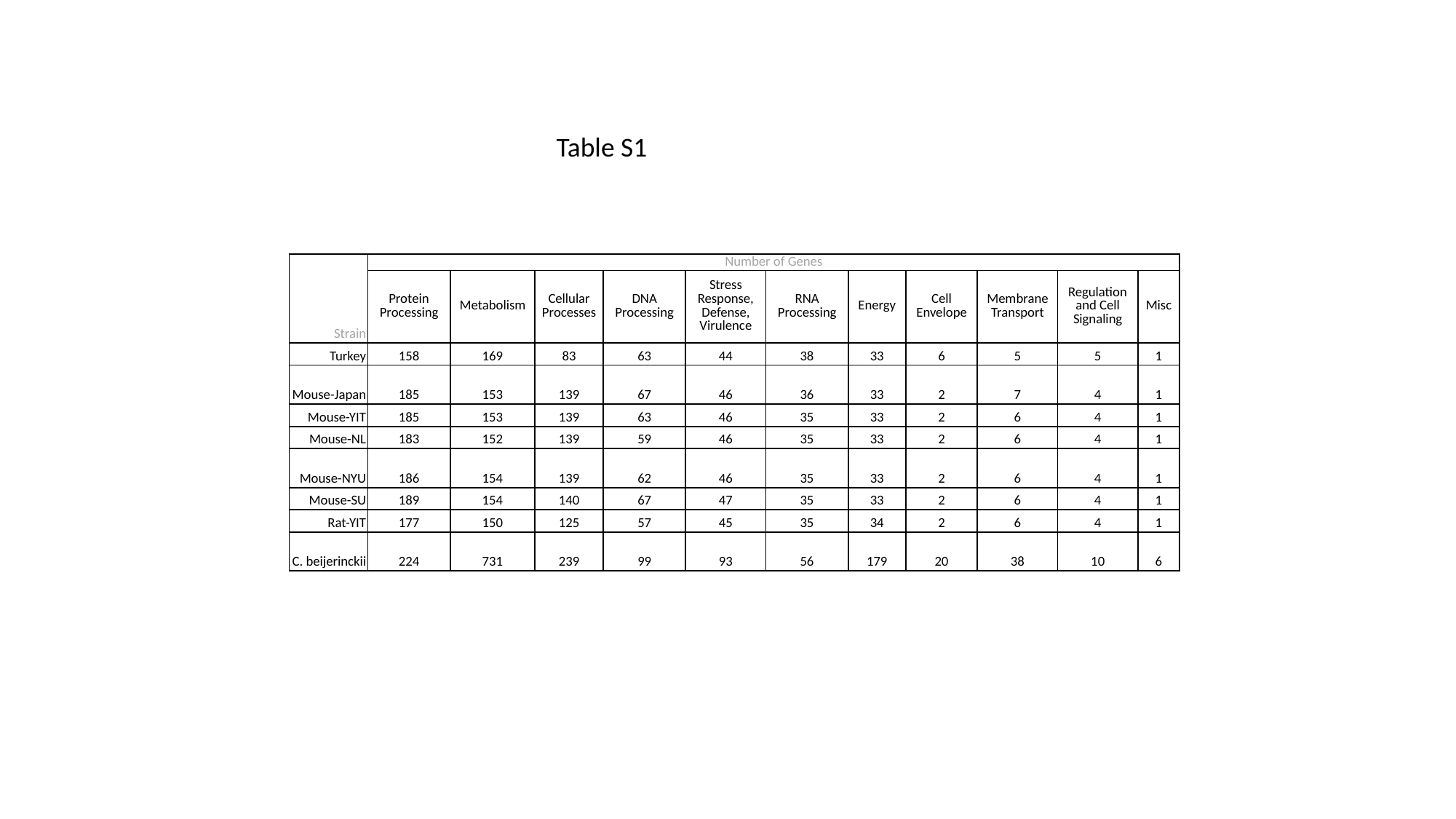

Table S1
| Strain | Number of Genes | | | | | | | | | | |
| --- | --- | --- | --- | --- | --- | --- | --- | --- | --- | --- | --- |
| | Protein Processing | Metabolism | Cellular Processes | DNA Processing | Stress Response, Defense, Virulence | RNA Processing | Energy | Cell Envelope | Membrane Transport | Regulation and Cell Signaling | Misc |
| Turkey | 158 | 169 | 83 | 63 | 44 | 38 | 33 | 6 | 5 | 5 | 1 |
| Mouse-Japan | 185 | 153 | 139 | 67 | 46 | 36 | 33 | 2 | 7 | 4 | 1 |
| Mouse-YIT | 185 | 153 | 139 | 63 | 46 | 35 | 33 | 2 | 6 | 4 | 1 |
| Mouse-NL | 183 | 152 | 139 | 59 | 46 | 35 | 33 | 2 | 6 | 4 | 1 |
| Mouse-NYU | 186 | 154 | 139 | 62 | 46 | 35 | 33 | 2 | 6 | 4 | 1 |
| Mouse-SU | 189 | 154 | 140 | 67 | 47 | 35 | 33 | 2 | 6 | 4 | 1 |
| Rat-YIT | 177 | 150 | 125 | 57 | 45 | 35 | 34 | 2 | 6 | 4 | 1 |
| C. beijerinckii | 224 | 731 | 239 | 99 | 93 | 56 | 179 | 20 | 38 | 10 | 6 |
