# Supplementary material for: Comparative genome analysis of commensal segmented filamentous bacteria (SFB) from turkey and murine hosts reveals distinct metabolic features
Source: BMC Genomics. 2022 Sep 17;23:659. doi: 10.1186/s12864-022-08886-x (PMC9482736; doi:10.1186/s12864-022-08886-x)
Supplement: Supplementary file 2 — Additional file 2: Figure S1. Averaged Subsystems Assignments of seven strains of segmented filamentous bacteria and Clostridium beijerinckii. PATRIC Subsystem assignments of the seven examined SFB strains and Clostridium beijerinckii as represented by the number of genes in each Subsystem category. Each of the 5 mouse-associated strains have been averaged and are represented as SFB-mouse (avg). [file 12864_2022_8886_MOESM2_ESM.pptx]

## Slide 1
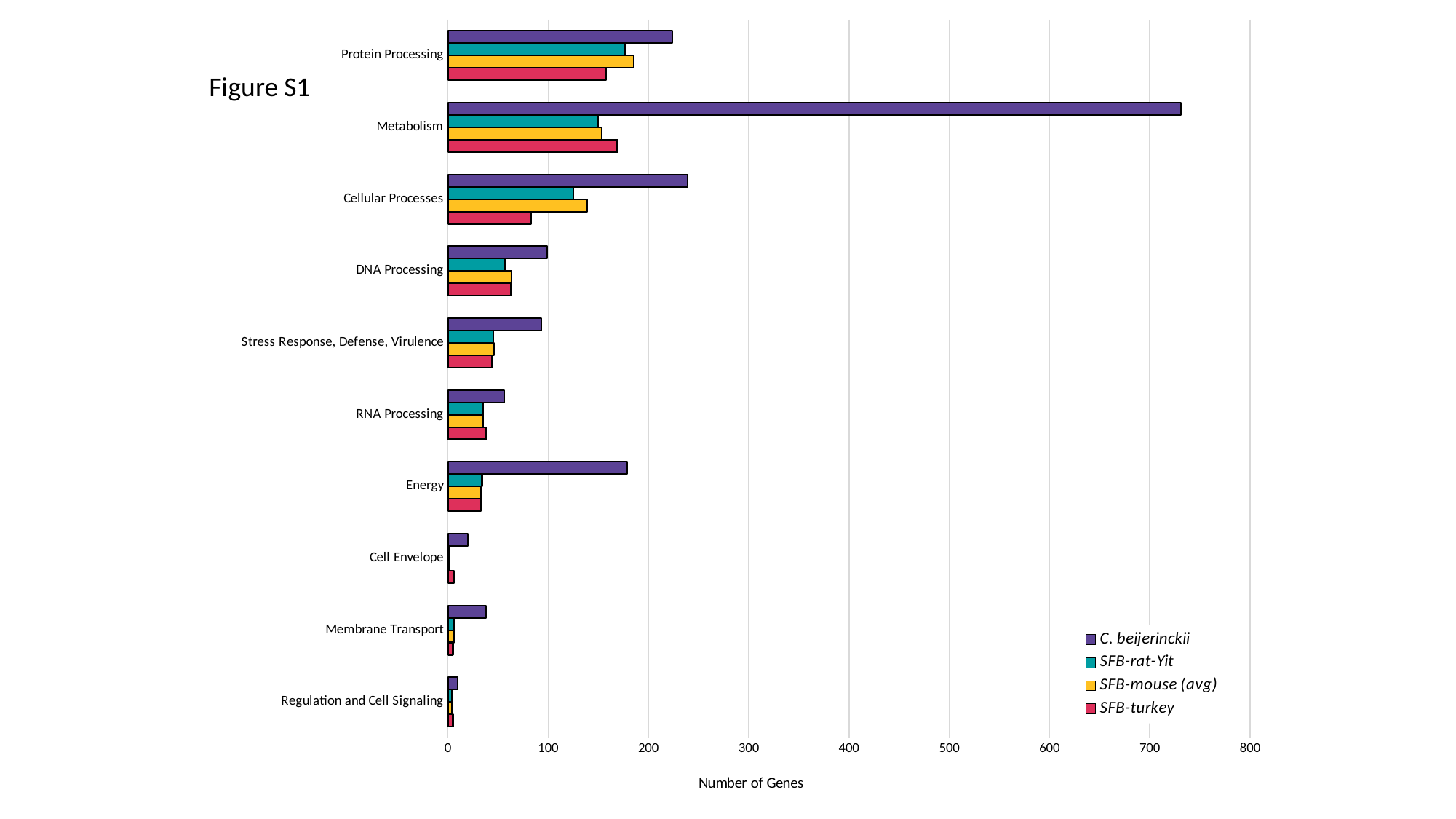

### Chart
| Category | SFB-turkey | SFB-mouse (avg) | SFB-rat-Yit | C. beijerinckii |
|---|---|---|---|---|
| Regulation and Cell Signaling | 5.0 | 4.0 | 4.0 | 10.0 |
| Membrane Transport | 5.0 | 6.2 | 6.0 | 38.0 |
| Cell Envelope | 6.0 | 2.0 | 2.0 | 20.0 |
| Energy | 33.0 | 33.0 | 34.0 | 179.0 |
| RNA Processing | 38.0 | 35.2 | 35.0 | 56.0 |
| Stress Response, Defense, Virulence | 44.0 | 46.2 | 45.0 | 93.0 |
| DNA Processing | 63.0 | 63.6 | 57.0 | 99.0 |
| Cellular Processes | 83.0 | 139.2 | 125.0 | 239.0 |
| Metabolism | 169.0 | 153.2 | 150.0 | 731.0 |
| Protein Processing | 158.0 | 185.6 | 177.0 | 224.0 |
