# Supplementary figures and images for: Comparative genome analysis of commensal segmented filamentous bacteria (SFB) from turkey and murine hosts reveals distinct metabolic features
Source: BMC Genomics. 2022 Sep 17;23:659. doi: 10.1186/s12864-022-08886-x (PMC9482736; doi:10.1186/s12864-022-08886-x)

## Slide 1
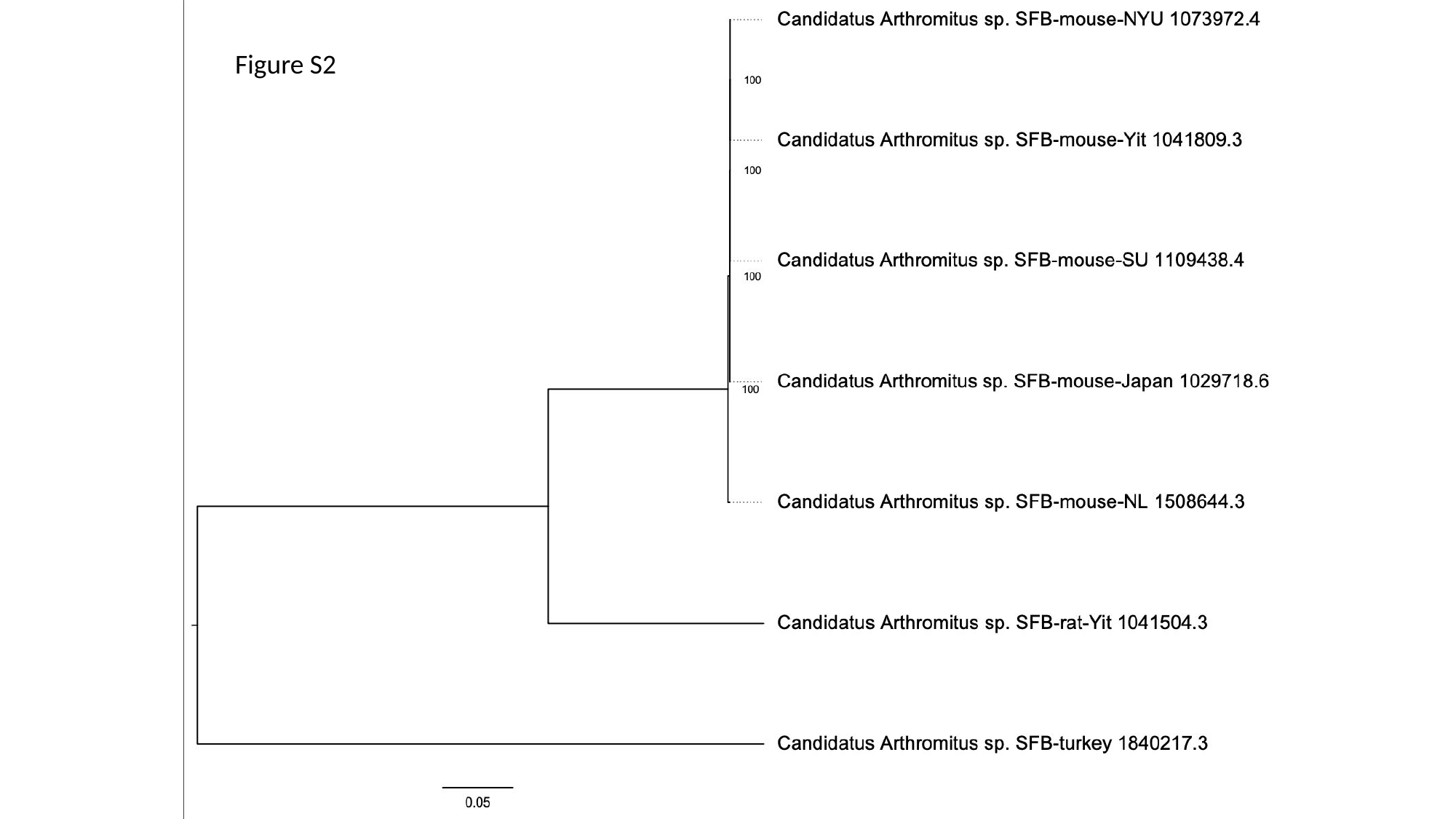

Figure S2

Supplement: Supplementary file 3 — Additional file 3: Figure S2. Phylogenetic Tree of seven SFB strains. Phylogenetic tree representing all the seven genomes of SFB strains. Tree was assembled using the PATRIC Codon Trees pipeline. [file 12864_2022_8886_MOESM3_ESM.pptx]
