# Supplementary material for: Comparative genome analysis of commensal segmented filamentous bacteria (SFB) from turkey and murine hosts reveals distinct metabolic features
Source: BMC Genomics. 2022 Sep 17;23:659. doi: 10.1186/s12864-022-08886-x (PMC9482736; doi:10.1186/s12864-022-08886-x)
Supplement: Supplementary file 4 — Additional file 4: Figure S3. Comparative Nucleotide Metabolism Pathways Between SFB-turkey and Murine SFB Strains. A schematic comparison of metabolic capabilities of SFB strains isolated from turkey and murine hosts to synthesize and degrade purines and pyrimidines. Color coding for divergent metabolism is as follows: enzymatic pathways shared by all the SFB strains appear in black, enzymatic pathways and transporters that are unique to SFB-turkey appear in purple, and complete metabolic pathways conserved in each strain appear in bold and italics. [file 12864_2022_8886_MOESM4_ESM.pptx]

## Slide 1
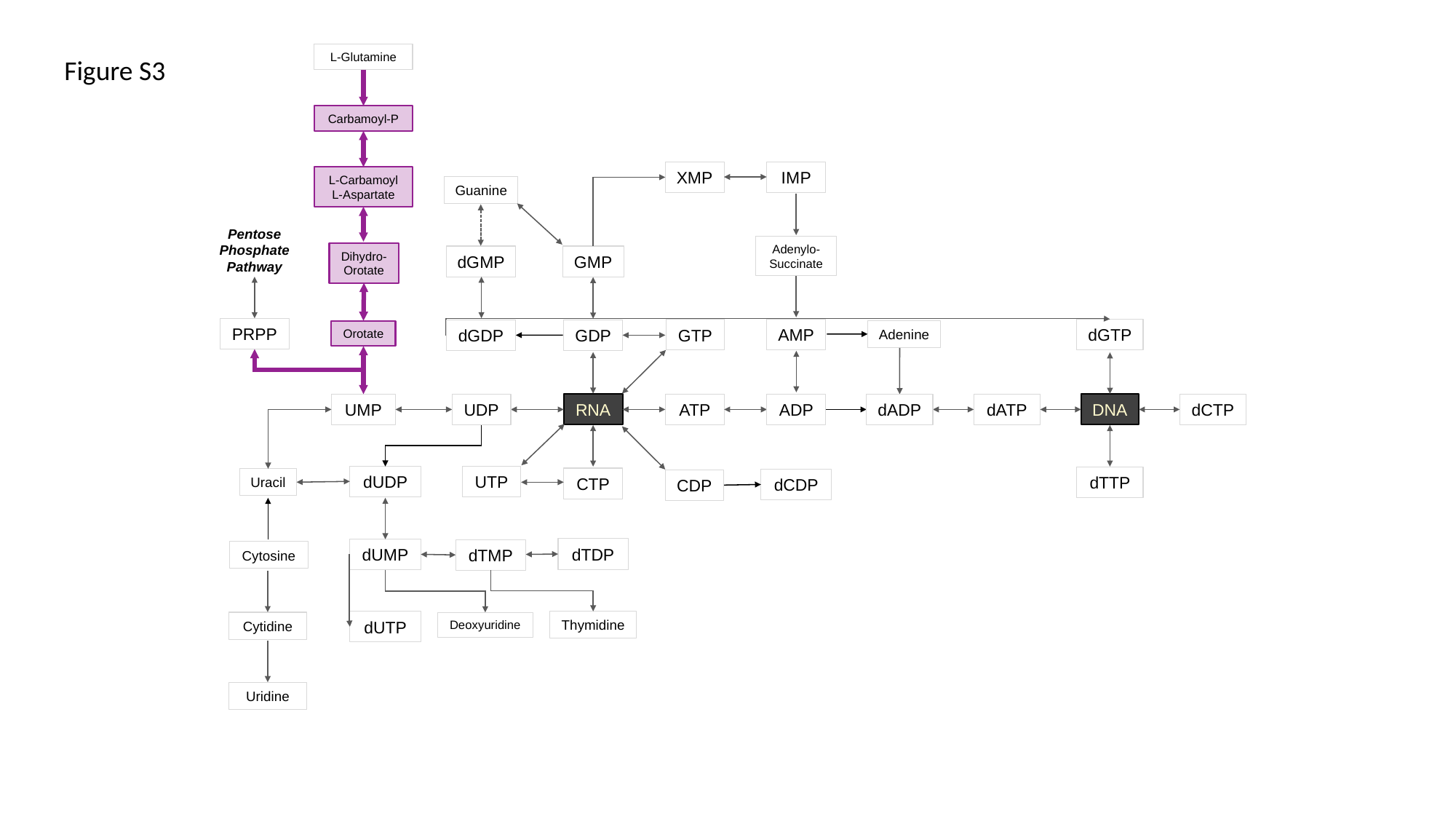

L-Glutamine
Figure S3
Carbamoyl-P
XMP
IMP
L-Carbamoyl L-Aspartate
Guanine
Pentose Phosphate Pathway
Adenylo- Succinate
Dihydro-
Orotate
dGMP
GMP
PRPP
AMP
dGTP
GTP
dGDP
GDP
Adenine
Orotate
UMP
UDP
RNA
ATP
ADP
dADP
dATP
DNA
dCTP
UTP
dUDP
dTTP
CTP
Uracil
dCDP
CDP
dTDP
dUMP
dTMP
Cytosine
dUTP
Thymidine
Cytidine
Deoxyuridine
Uridine
